# Is my body better than yours? Validation of the German version of the Upward and Downward Physical Appearance Comparison Scales in individuals with and without eating disorders

**DOI:** 10.3389/fpsyg.2024.1390063

**Published:** 2024-05-28

**Authors:** Kristine Schönhals, Hannah L. Quittkat, Mona M. Voges, Gritt Ladwig, Friederike-Johanna Holtmann, Silja Vocks

**Affiliations:** Department of Clinical Psychology and Psychotherapy, Institute of Psychology, Osnabrück University, Osnabrück, Germany

**Keywords:** social comparison, upward physical appearance comparison, downward physical appearance comparison, validation, eating disorders, body image, body dissatisfaction

## Abstract

**Introduction:**

This study examines the psychometric properties of a German version of the Upward and Downward Physical Appearance Comparison Scales (UPACS and DACS).

**Methods:**

A total of 2,114 participants, consisting of 1,360 women without eating disorders (*M*_age_ = 25.73, *SD*_age_ = 6.84), 304 men without eating disorders (*M*_age_ = 24.48, *SD*_age_ = 6.34), and 450 women with eating disorders (*M*_age_ = 27.11, *SD*_age_ = 7.21), completed the UPACS and DACS as well as further questionnaires on appearance comparisons, eating disorder pathology, and self-esteem.

**Results:**

Structural equation modeling confirmed the proposed one-factor structure of the original English-language version of the DACS but not of the UPACS. Both scales showed good internal consistency and test–retest reliability. The UPACS and DACS showed the expected correlations with related constructs, indicating acceptable construct validity, with some limitations for women with eating disorders.

**Discussion:**

Overall, this study indicates that the German versions of the UPACS and DACS are psychometrically suitable for assessing upward and downward physical appearance comparisons in women and men without eating disorders and women with eating disorders in research and clinical practice.

## 1 Introduction

Body dissatisfaction is a risk factor for the development of eating disorders (EDs, Grabe et al., 2008; Rohde et al., 2015) and is strongly associated with appearance comparisons: The tripartite influence model of body image and eating disturbance proposes that comparing one’s appearance to that of others mediates the influence of peers, parents, and media on body dissatisfaction (Thompson et al., 1999). In line with this, girls and women with ED symptoms have been found to engage more frequently in appearance comparisons than women without ED symptoms (Corning et al., 2006; Hamel et al., 2012). Moreover, the association between appearance comparisons and body dissatisfaction is assumed to be stronger in women than in men (Myers and Crowther, 2009), and research shows that women are more likely to engage in such comparisons (Strahan et al., 2006).

Adapted from Festinger (1954), appearance comparisons can take the form of upward comparisons, in which others are perceived as more attractive than oneself, or downward comparisons, in which others are perceived as less attractive. Upward appearance comparisons have frequently been related to lower self-esteem (Schmuck et al., 2019; Rüther et al., 2023), disordered eating (Blechert et al., 2009; Arigo et al., 2014), negative mood, and body dissatisfaction (Leahey et al., 2007, 2011; Ridolfi et al., 2011; Myers et al., 2012). The associations and effects of downward appearance comparisons, by contrast, are less clear. While many studies have reported associations of downward appearance comparisons with increased self-esteem (Pan and Peña, 2020), positive mood, and body satisfaction (van den Berg and Thompson, 2007; Bailey and Ricciardelli, 2010), others suggested that downward appearance comparisons do not have protective effects against body dissatisfaction and eating pathology (Fitzsimmons-Craft, 2017; Rogers et al., 2017), or even found associations with higher levels of body dissatisfaction (Vartanian and Dey, 2013), disordered eating (Drutschinin et al., 2018), drive for thinness, and restrained eating among women (Lin and Soby, 2016). The latter effect might be explained by the consideration that in general, downward comparisons are more commonly used as a coping strategy by individuals with low self-esteem (Wills, 1981). Additionally, the tendency to make upward appearance comparisons and the tendency to make downward appearance comparisons are associated with one another (O’Brien et al., 2009), indicating that people who engage in appearance comparisons do so in both directions, although upward appearance comparisons are often more prevalent (Ridolfi et al., 2011; McCarthy et al., 2023).

To assess general appearance comparisons in German-speaking individuals, the German version of the Physical Appearance Comparison Scale (PACS, original version: Thompson et al., 1991) is available and has been validated for women and men without EDs and women with anorexia nervosa (Mölbert et al., 2017). However, the PACS does not differentiate between upward and downward appearance comparisons. To overcome this limitation, O’Brien et al. (2009) developed the English-language Upward and Downward Physical Appearance Comparison Scales (UPACS and DACS). The UPACS and DACS are short and therefore economic scales consisting of 10 and eight items, respectively, with good internal reliability and construct validity. Principal component analysis revealed a one-factor solution for each scale. To the best of our knowledge, to date, there is no validated German-language questionnaire for the assessment of upward and downward appearance comparisons.

Therefore, the aim of the present study was to translate the UPACS and DACS into German and to examine the psychometric properties of the translated versions of both scales in a sample of women and men without EDs and women with EDs. We expected that women without EDs would show higher scores on the UPACS and DACS than men without EDs, as women tend to engage in appearance comparisons to a greater extent than men (Davison and McCabe, 2005; Strahan et al., 2006; O’Brien et al., 2009). Furthermore, we assumed that women with EDs would show higher scores on both scales than women without EDs, as appearance comparisons play a crucial role in the development of body dissatisfaction, which is in turn associated with EDs (Thompson et al., 1999; Leahey et al., 2011). Consistent with the original versions of the scales, we hypothesized a one-factor structure for both the UPACS and the DACS for all examined subsamples (O’Brien et al., 2009). In terms of construct validity, when examining the correlations of the UPACS and DACS with related measures, we assumed (1) positive correlations with established questionnaires regarding general physical appearance comparisons and eating pathology, in line with the original research (O’Brien et al., 2009), and (2) negative correlations with self-esteem, given that upward appearance comparisons have been found to be harmful for self-esteem (Schmuck et al., 2019), while downward comparisons are more frequently used as a coping mechanism by individuals with low self-esteem (Wills, 1981). Overall, from a descriptive perspective, we postulated stronger effects for the UPACS than for the DACS, as upward appearance comparisons have been more conclusively related to body image disturbances.

## 2 Materials and methods

### 2.1 Participants

The sample for the present study consisted of *N* = 2,114 participants aged between 18 and 78 years (*M*_age_ = 25.84, *SD*_age_ = 6.89) who completed the UPACS and DACS and related measures across nine studies. The sample comprised *n* = 1,360 women without eating disorders (*M*_age_ = 25.73, *SD*_age_ = 6.84), *n* = 304 men without eating disorders (*M*_age_ = 24.48, *SD*_age_ = 6.34), and *n* = 450 women with eating disorders (*M*_age_ = 27.11, *SD*_age_ = 7.21, *n* = 191 anorexia nervosa, *n* = 132 bulimia nervosa, *n* = 127 binge eating disorder). Across the studies, *n* = 338 diagnoses were self-reported and *n* = 112 diagnoses were assessed using a structured clinical interview within the respective study (Structured Clinical Interview for DSM-IV, Wittchen et al., 1997; or Diagnostic Interview for Mental Disorders, Margraf et al., 2017). Some participants who took part in one of the nine studies were not included in the final sample: e.g., one man, who was the only man to self-report an ED, meaning that it was not possible to analyze a subsample of men with EDs; 117 participants with other or unclear EDs; nine participants with implausible or missing data (e.g., values outside the range of the respective scales); and seven participants under the age of 18 years. Participants’ body mass index (BMI) ranged from 12.89 kg/m^2^ to 65.31 kg/m^2^ (*M*_BMI_ = 23.68, *SD*_BMI_ = 6.37). Across all participants, 9.46% were underweight (BMI < 18.50), 66.65% were of normal weight (BMI 18.50–24.99), 12.16% were overweight (BMI 25.00–29.99), and 11.73% were obese (BMI > 30.00). The characteristics of the subsamples of the different studies are displayed in Table 1.

**Table 1:** Sample characteristics within the different studies.

Study	*n* included in this study^a^	EDs^b^	ED assessment	BMI in kg/m^2^	Age	UPACS	DACS
1. Voges et al. (2018)	*n* = 186 women (8.80%)	*n* = 33 AN *n =* 30 BN *n =* 9 BED	SCID-I	*Rg =* 12.89–45.17 *M* = 21.18 *SD* = 4.76	*Rg =* 18–34 *M* = 21.99 *SD* = 3.44	*Rg =* 1.00–5.00 *M* = 3.47 *SD* = 0.83	*Rg =* 1.00–4.75 *M* = 2.39 *SD* = 0.93
2. Voges et al. (2019)	*n* = 109 men (5.16%)	None		*Rg =* 18.59–26.88 *M* = 22.84 *SD* = 1.61	*Rg =* 18–31 *M* = 23.13 *SD* = 3.07	*Rg =* 1.00–4.80 *M* = 2.86 *SD* = 0.88	*Rg =* 1.00–5.00 *M* = 2.18 *SD* = 0.90
3. Voges et al. (2020)	*n* = 53 women (2.51%)	None		*Rg =* 19.05–22.76 *M* = 20.69 *SD* = 1.06	*Rg =* 18–28 *M* = 22.09 *SD* = 2.48	*Rg =* 1.00–5.00 *M* = 3.28 *SD* = 1.02	*Rg =* 1.00–4.13 *M* = 2.17 *SD* = 0.91
4. Voges et al. (2022)	*n* = 64 women *n* = 64 men (6.05%)	None		*Rg =* 17.59–30.64 *M* = 22.37 *SD* = 2.73	*Rg =* 18–30 *M* = 22.62 *SD* = 2.89	*Rg =* 1.00–4.90 *M* = 3.23 *SD* = 0.92	*Rg =* 1.00–4.00 *M* = 2.18 *SD* = 0.88
5. Quittkat et al.^c^	*n* = 58 women *n* = 53 men (5.25%)	None		*Rg =* 18.72–29.48 *M* = 22.93 *SD* = 2.50	*Rg =* 18–27 *M* = 22.41 *SD* = 2.18	*Rg =* 1.00–4.80 *M* = 3.21 *SD* = 0.91	*Rg =* 1.00–4.13 *M* = 2.30 *SD* = 0.86
6. Quittkat et al. (2023)	*n* = 120 women (5.68%)	*n* = 40 BED	DIPS	*Rg =* 18.71–61.56 *M* = 33.53 *SD* = 10.99	*Rg =* 22–49 *M* = 32.15 *SD* = 6.02	*Rg =* 1.00–4.90 *M* = 3.00 *SD* = 0.96	*Rg =* 1.00–5.00 *M* = 2.65 *SD* = 1.03
7. Ladwig et al. (2024)^d^	*n* = 651 women (30.79%)	*n* = 101 AN *n =* 66 BN *n =* 47 BED	Self-report	*Rg =* 13.38–65.31 *M* = 24.09 *SD* = 6.81	*Rg =* 18–63 *M* = 28.48 *SD* = 7.43	*Rg =* 1.00–5.00 *M* = 3.54 *SD* = 0.88	*Rg =* 1.00–5.00 *M* = 2.48 *SD* = 0.95
8. Holtmann et al. (2024)^e^	*n* = 396 women (18.73%)	*n* = 34 AN *n =* 23 BN *n =* 17 BED	Self-report	*Rg =* 13.96–49.47 *M* = 22.51 *SD* = 5.09	*Rg =* 18–78 *M* = 23.75 *SD* = 5.82	*Rg =* 1.00–5.00 *M* = 3.51 *SD* = 0.88	*Rg =* 1.00–5.00 *M* = 2.43 *SD* = 0.97
9. Present study	*n* = 282 women *n* = 78 men (17.03%)	*n* = 23 AN *n =* 13 BN *n =* 14 BED	Self-report	*Rg =* 14.20–62.10 *M* = 23.63 *SD* = 5.40	*Rg =* 18–68 *M* = 26.86 *SD* = 8.08	*Rg =* 1.00–5.00 *M* = 3.18 *SD* = 0.98	*Rg =* 1.00–5.00 *M* = 2.31 *SD* = 0.98

ED, eating disorder; BMI, body mass index; UPACS, Upward Physical Appearance Comparison Scale; DACS, Downward Physical Appearance Comparison Scale; AN, anorexia nervosa; BN, bulimia nervosa; BED, binge eating disorder; SCID-I, Structured Clinical Interview for DSM-IV; DIPS, Diagnostic Interview for Mental Disorders; Rg, range; M, mean; SD, standard deviation.

a Percentage in parentheses: Proportion of the sample forming the whole sample (*N* = 2,114).

b Only women.

c Unpublished data.

d Manuscript submitted for publication.

e Manuscript in preparation.

### 2.2 Measures

#### 2.2.1 Upward and Downward Physical Appearance Comparison Scales (UPACS and DACS)

The UPACS consists of 10 items assessing upward physical appearance comparisons and the DACS consists of eight items assessing downward physical appearance comparisons (O’Brien et al., 2009). For both scales, items are rated on a 5-point Likert scale from *1* = *strongly disagree* to *5 = strongly agree*, with higher scores indicating a higher tendency for upward or downward physical appearance comparisons, respectively. In the original study, Cronbach’s alpha was α = 0.93 (women: α = 0.94, men: α = 0.91) for the UPACS and α = 0.90 (women: α = 0.90, men: α = 0.89) for the DACS, and the two-week test–retest reliability was *r*_tt_ = 0.79 (UPACS) and *r*_tt_ = 0.70 (DACS). The English versions of the scales were translated into German by German-speaking members of the Department of Clinical Psychology and Psychotherapy of Osnabrück University (see Supplementary material). No back-translation process was administered; however, a bilingual translator compared the two versions of the scales and found only three minor expressions that could have been adapted but do not alter the content of the items.

#### 2.2.2 Physical Appearance Comparison Scale

The PACS (Thompson et al., 1991) assesses the frequency of general appearance comparisons. The German version of the scale consists of five items that are rated on a 5-point Likert scale (*1* = *never* to *5 = always*, Mölbert et al., 2017), with higher scores indicating a higher tendency for general physical appearance comparisons. The internal consistency for the current sample was acceptable, at McDonald’s ω_t_ = 0.84.

#### 2.2.3 Eating Disorder Examination Questionnaire

The EDE-Q assesses the psychopathology of EDs (Fairburn and Beglin, 1994). The German version used in the present study encompasses 22 items across the four subscales *Restraint, Eating concern, Weight concern* and *Shape concern.* Items are rated on a 6-point Likert scale based on the frequency of certain behaviors in the past 28 days (*0* = *no day* to *6 = every day*) or the severity of the behaviors (*0* = *not at all* to *6 = very much,* Hilbert and Tuschen-Caffier, 2016). Higher scores indicate higher ED pathology. The internal consistency for the current sample was acceptable, at McDonald’s ω_t_ = 0.98 for the global score across the four subscales.

#### 2.2.4 Eating Disorder Inventory-2

The EDI-2 assesses ED-related characteristics (Garner, 1991). The two subscales *Drive for thinness* and *Body dissatisfaction* of the German version used in the present study contain 16 items combined, rated on a 6-point Likert scale (*1* = *never* to *6 = always,* Paul and Thiel, 2005), with higher scores indicating a higher level of drive for thinness and body dissatisfaction, respectively. The internal consistencies for the current sample were acceptable, at McDonald’s ω_t_ = 0.95 for the *Drive for thinness* subscale, and McDonald’s ω_t_ = 0.95 for the *Body dissatisfaction* subscale.

#### 2.2.5 Rosenberg Self-Esteem Scale

The RSES assesses general self-esteem (Rosenberg, 1965). The German version contains 10 items rated on a 4-point Likert scale (*0* = *strongly disagree* to *3 = strongly agree*, Ferring and Filipp, 1996), with higher scores indicating higher self-esteem. The internal consistency for the current sample was acceptable, at McDonald’s ω_t_ = 0.95.

### 2.3 Procedure

As the German-language versions of the UPACS and DACS were part of several different studies conducted at the Department of Clinical Psychology and Psychotherapy of Osnabrück University, data were available from eight studies conducted between 2016 and 2023 [Study 1: Voges et al., 2018; Study 2: Voges et al., 2019; Study 3: Voges et al., 2020; Study 4: Voges et al., 2022; Study 5: unpublished data; Study 6: Quittkat et al., 2023; Study 7: Ladwig et al., 2024 (manuscript submitted for publication); Study 8: Holtmann et al., 2024 (manuscript in preparation)]. Study 9 was performed in order to obtain additional data, particularly regarding convergent validity with the PACS, to ensure an appropriate sample size for men, and to examine the test–retest reliability. The ninth study was the only study in which men with eating disorders or non-binary people could have participated. Unfortunately, only one man with an eating disorder and no non-binary people participated; therefore, no analyses for these groups could be conducted. In the nine studies, data were collected using either an online survey presented in Unipark or LimeSurvey, or through paper-and-pencil questionnaires. Participants were required to be at least 18 years old and were primarily recruited through email distribution lists, flyers, social media posts (e.g., on Instagram), and cooperation with clinics.

In all nine studies, participants provided sociodemographic information and completed the UPACS and DACS, EDE-Q, EDI-2, and RSES. The PACS was administered in Studies 8 and 9 only. Participants who completed the questionnaires in Study 9 received an email asking them to complete the UPACS and DACS again 2 weeks later, and were reminded to take part in the retest a further week later. In total, *n* = 232 participants (*n* = 142 women without EDs, *n* = 50 men without EDs, *n* = 40 women with EDs) completed both assessments.

For the sample of women with EDs, EDs were either self-reported by participants or assessed using a structured clinical interview. While Study 1 used the Structured Clinical Interview for DSM-IV (SCID-I, Wittchen et al., 1997), Study 6 examined EDs using the Diagnostic Interview for Mental Disorders (DIPS, Margraf et al., 2017).

### 2.4 Data analysis

Descriptive data, comparisons between groups, and correlation analyses were calculated using IBM SPSS (version 28.0.1.1). McDonald’s ω_t_ was calculated using the *psych* package in R (version 4.3.2). Structural equation modeling (SEM) was performed using the *lavaan* package in R. As the assumption of normality was violated (Kolmogorov–Smirnov tests with Lilliefors correction, *p* < 0.001 for each respective scale in every subsample), Mann–Whitney U tests were performed to compare (a) the scores on the EDI-2 subscales and the EDE-Q global score between women with self-reported EDs and women with EDs diagnosed within a respective study, and (b) the UPACS and DACS scores between women and men without EDs and between women with and without EDs. All further analyses were conducted separately for (1) women without EDs, (2) men without EDs, and (3) women with EDs. The internal consistency was considered acceptable at McDonald’s ω_t_ > 0.65 (Kalkbrenner, 2023). SEM with maximum likelihood estimation was used to examine whether the one-factor structure of the original scales can be transferred to the German versions. The fit/misfit indices χ^2^, CFI, NNFI, RMSEA, and SRMR were determined to assess the goodness of fit of the one-factor models. The thresholds for good fit were CFI ≥ 0.97, NNFI ≥0.97, RMSEA ≤0.05 and SRMR ≤0.05, and the thresholds for acceptable fit were CFI ≥ 0.95, NNFI ≥0.95, RMSEA ≤0.08 and SRMR ≤0.10 (adapted from West et al., 2012). In the case of a poor model fit, possible re-specifications were examined through modification indices, solely for exploratory purposes in order to discuss possible future adaptations of the scales. Furthermore, measurement invariance across all subsamples was examined. Following Putnick and Bornstein (2016), measurement invariance was tested in four steps: (1) configural invariance, (2) metric invariance (loading invariance), (3) scalar invariance (intercept invariance) and (4) residual invariance. Invariance was defined based on the recommendations by Chen (2007). Thresholds that indicated non-invariance were ΔCFI ≤ −0.005, ΔRMSEA ≥0.010, and ΔSRMR ≥0.025 for metric invariance and ΔSRMR ≥0.005 for scalar and residual invariance. As tests of correlations against 0 are robust to non-normality (Fowler, 1987), Pearson correlations were used to assess test–retest reliability and correlations with related constructs. The test–retest reliability was calculated by correlating the first and second assessments of the UPACS and DACS. To examine construct validity, the PACS, the subscales of the EDI-2, the EDE-Q, and the RSES were correlated with the UPACS and DACS. Furthermore, the UPACS and DACS were correlated with each other. Bonferroni correction was applied for the correlations regarding construct validity within each subsample, thus correcting for 11 significance tests in each case.

## 3 Results

### 3.1 Preliminary analyses

When comparing women with self-reported EDs and women with EDs diagnosed within one of the studies, no significant differences emerged regarding the *Body dissatisfaction* subscale of the EDI-2, *z* = 0.48, *p* = 0.64. However, the two groups differed significantly on the *Drive for thinness* subscale of the EDI-2, *z* = 3.34, *p* < 0.001, and on the EDE-Q, *z* = 3.69, *p* < 0.001, with women with self-reported EDs showing significantly higher levels of ED pathology. In order to include a greater range and variability of ED pathology, the further analyses include both women with diagnosed and self-reported EDs.

As expected, women without EDs showed significantly higher scores on the UPACS than men without EDs, *z* = 6.75, *p* < 0.001, and significantly lower scores than women with EDs, *z* = 12.21, *p* < 0.001. Likewise, women without EDs showed significantly higher scores on the DACS than men without EDs, *z* = 4.56, *p* < 0.001, and significantly lower scores than women with EDs, *z* = 6.66, *p* < 0.001.

The corrected item-total correlations (all ≥ 0.50) for the UPACS and DACS items were good in every subsample, indicating that all items correlate sufficiently with each respective scale (see Tables 2, 3). None of the items were normally distributed, as indicated by Kolmogorov–Smirnov tests with Lilliefors correction (all *p* < 0.001). Skewness and kurtosis of all items as well as box plots for the UPACS and DACS can be derived from the Supplementary material.

**Table 2:** Means, standard deviations, and corrected item-total correlations for the UPACS in different subsamples.

UPACS items	Women without EDs (*n* = 1,360)			Men without EDs (*n* = 304)			Women with EDs (*n* = 450)		
	*M*	*SD*	Corrected item-total correlation	*MD*	*SD*	Corrected item-total correlation	*M*	*SD*	Corrected item-total correlation
1. I compare myself to those who are better looking than me rather than those who are not.	3.68	1.02	0.58	3.45	1.05	0.62	4.21	0.90	0.50
2. I tend to compare my own physical attractiveness to that of magazine models.	2.35	1.27	0.54	1.87	1.10	0.50	2.98	1.28	0.53
3. I find myself thinking about whether my own appearance compares well with models and movie stars.	2.52	1.28	0.55	2.22	1.22	0.55	2.89	1.37	0.50
4. At the beach or athletic events (sports, gym, etc.) I wonder if my body is as attractive as the people I see there with very attractive bodies.	3.30	1.25	0.68	3.00	1.20	0.70	3.62	1.30	0.53
5. I tend to compare myself to people I think look better than me.	3.65	1.08	0.79	3.20	1.19	0.81	4.22	0.95	0.66
6. When I see a person with a great body, I tend to wonder how I ‘match up’ with them.	3.46	1.16	0.78	3.06	1.27	0.83	4.15	1.01	0.71
7. When I see good-looking people I wonder how I compare to them.	3.49	1.14	0.82	3.03	1.20	0.85	4.12	0.98	0.75
8. At parties or other social events, I compare my physical appearance to the physical appearance of the very attractive people.	3.38	1.21	0.81	2.87	1.24	0.83	4.00	1.07	0.74
9. I find myself comparing my appearance with people who are better looking than me.	3.56	1.09	0.84	3.11	1.20	0.85	4.14	0.96	0.77
10. I compare my body to people who have a better body than me.	3.58	1.12	0.83	3.21	1.22	0.82	4.20	0.92	0.79

UPACS, Upward Physical Appearance Comparison Scale; ED, eating disorder; M, mean; SD, standard deviation.

**Table 3:** Means, standard deviations and corrected item-total correlations for the DACS in different subsamples.

DACS items	Women without EDs (*n* = 1,360)			Men without EDs (*n* = 304)			Women with EDs (*n* = 450)		
	*M*	*SD*	Corrected item-total correlation	*M*	*SD*	Corrected item-total correlation	*M*	*SD*	Corrected item-total correlation
1. When I see a person who is physically unattractive I think about how my body compares to theirs.	2.86	1.26	0.69	2.54	1.27	0.73	3.42	1.29	0.60
2. I tend to compare my body to those who have below average bodies.	2.26	1.10	0.69	1.98	1.03	0.76	2.75	1.25	0.55
3. At the beach, gym, or sporting events I compare my body to those with less athletic bodies.	2.49	1.19	0.69	2.21	1.08	0.66	2.73	1.27	0.64
4. I compare myself to people less good looking than me.	2.39	1.11	0.82	2.07	0.98	0.83	2.55	1.20	0.79
5. I think about how attractive my body is compared to overweight people.	2.39	1.27	0.69	2.08	1.15	0.62	2.90	1.37	0.64
6. At parties I often compare my looks to the looks of unattractive people.	2.04	1.06	0.79	1.88	0.95	0.79	2.41	1.22	0.80
7. I often compare myself to those who are less physically attractive.	2.16	1.09	0.85	1.92	0.99	0.85	2.48	1.27	0.83
8. I tend to compare my physical appearance with people whose bodies are not as physically appealing.	2.23	1.11	0.84	1.98	1.02	0.83	2.50	1.21	0.84

DACS, Downward Physical Appearance Comparison Scale; ED, eating disorder; M, mean; SD, standard deviation.

### 3.2 Confirmatory factor analyses

The results of the SEM analyses for the different subsamples are displayed in Table 4. Regarding the UPACS, the one-factor structure showed a poor fit on all examined indices, with the exception of the SRMR for all subsamples, which indicated an acceptable fit. Across all subsamples, the standardized loadings of the items were 0.46 ≤ λ* ≤ 0.92 (see Table 5). Invariance testing did not support configural invariance in the CFI and RMSEA; only the SRMR indicated an acceptable fit. Therefore, the further steps are not reported, but can be derived from Table 6. In sum, these results indicate that the proposed one-factor structure of the German UPACS is not adequate for all subsamples. For exploratory purposes, we examined re-specifications based on modification indices. In all subsamples, modification indices indicated a substantial improvement of the model fit when allowing for correlations of the errors of the two items “I find myself thinking about whether my own appearance compares well with models and movie stars” and “I tend to compare my own physical attractiveness to that of magazine models” (see Table 4, all MI > 90), indicating a redundancy of the item contents (Byrne, 2016). Despite the poor fit, the further analyses were nevertheless conducted for the entire 10-item UPACS in order to examine the psychometric properties in parallel to the original version, and because the good item-total correlations still indicate sufficient relations of each item with the rest of the scale.

**Table 4:** Structural equation modeling for the UPACS and DACS in different subsamples.

Model	MI	χ^2^	*df*	CFI	NNFI	RMSEA [90% *CI*]	SRMR
Women without EDs (*n* = 1,360)							
UPACS		883.25^***^	35	0.918	0.895	0.133 [0.126, 0.141]	0.060
UPACS mod. Items 2 and 3	320.67	523.42^***^	34	0.953	0.938	0.103 [0.095, 0.111]	0.036
DACS		202.70^***^	20	0.977	0.967	0.082 [0.072, 0.092]	0.024
Men without EDs (*n* = 304)							
UPACS		241.09^***^	35	0.915	0.890	0.139 [0.123, 0.156]	0.060
UPACS mod. Items 2 and 3	74.42	157.14^***^	34	0.949	0.933	0.109 [0.092, 0.127]	0.034
DACS		68.83^***^	20	0.973	0.962	0.090 [0.067, 0.113]	0.033
Women with EDs (*n* = 450)							
UPACS		287.18^***^	35	0.899	0.871	0.127 [0.113, 0.140]	0.064
UPACS mod. Items 2 and 3	94.64	183.13^***^	34	0.941	0.921	0.099 [0.085, 0.113]	0.044
DACS		90.35^***^	20	0.970	0.957	0.088 [0.070, 0.107]	0.035

Structural equation modeling with maximum likelihood estimation, including re-specification analysis for the UPACS, in which suggestions are made to allow for correlation of errors between items. The suggestion with the highest MI in each subsample is reported. UPACS, Upward Physical Appearance Comparison Scale; UPACS mod., re-specification analysis for the UPACS; DACS, Downward Physical Appearance Comparison Scale; CFI, comparative fit index; NNFI, non-normed fit index; RMSEA, root mean square error of approximation; CI, confidence interval; SRMR, standardized root mean square residual; ED, eating disorder. The specific items can be derived from Tables 2, 3. ^*^p < 0.05, ^**^p < 0.01, ^***^p < 0.001.

**Table 5:** Standardized loadings for structural equation modeling for the UPACS in different subsamples.

UPACS item	Standardized loadings λ* for women without EDs (*n* = 1,360)	Standardized loadings λ* for men without EDs (*n* = 304)	Standardized loadings λ* for women with EDs (*n* = 450)
Item 1	0.61	0.65	0.56
Item 2	0.49	0.47	0.49
Item 3	0.50	0.53	0.46
Item 4	0.68	0.72	0.54
Item 5	0.84	0.84	0.73
Item 6	0.81	0.85	0.75
Item 7	0.86	0.89	0.79
Item 8	0.86	0.87	0.82
Item 9	0.92	0.91	0.87
Item 10	0.90	0.87	0.87

Structural equation modeling with maximum likelihood estimation. UPACS, Upward Physical Appearance Comparison Scale. ED, eating disorder. The specific items can be derived from Table 2.

**Table 6:** Measurement invariance across all subsamples.

Model	χ^2^	Δχ^2^	*df*	Δ*df*	CFI	ΔCFI	RMSEA [90% *CI*]	ΔRMSEA	SRMR	ΔSRMR
UPACS										
Configural	1411.52^***^		105		0.915		0.133 [0.127, 0.139]		0.056	
Metric	1436.89^***^	25.37	123	18	0.914	−0.001	0.123 [0.117, 0.129]	−0.010	0.062	0.006
Scalar	1512.51^***^	75.62^***^	141	18	0.910	−0.004	0.117 [0.112, 0.123]	−0.006	0.065	0.003
Residual	1609.19^***^	96.68^***^	161	20	0.905	−0.005	0.113 [0.108, 0.118]	−0.004	0.069	0.004
DACS										
Configural	361.88^***^		60		0.975		0.084 [0.076, 0.093]		0.025	
Metric	416.17^***^	54.29^***^	74	14	0.971	−0.004	0.081 [0.074, 0.089]	−0.003	0.044	0.019
Scalar	527.63^***^	111.46^***^	88	14	0.963	−0.008	0.084 [0.077, 0.091]	0.003	0.048	0.004
Residual	749.27^**^	221.64^***^	104	16	0.946	−0.017	0.094 [0.088, 0.100]	0.010	0.051	0.003

Test of measurement invariance via structural equation modeling with maximum likelihood estimation. Differences Δ indicate the change in the respective index between two consecutive steps. UPACS, Upward Physical Appearance Comparison Scale; DACS, Downward Physical Appearance Comparison Scale; CFI, comparative fit index; NNFI, non-normed fit index; RMSEA, root mean square error of approximation; CI, confidence interval; SRMR, standardized root mean square residual; ED, eating disorder.

^**^p < 0.01, ^***^p < 0.001.

Concerning the DACS, for all subsamples, the one-factor structure showed a good fit according to the CFI and SRMR, an acceptable fit according to the NNFI, and a mediocre fit according to the RMSEA. Across all subsamples, the standardized loadings of the items lay at 0.55 ≤ λ* ≤ 0.91 (see Table 7). Tests of measurement invariance supported configural and metric invariance (see Table 6). For scalar and residual invariance, the changes in the CFI lay above the threshold, indicating non-invariance, but the changes in the RMSEA and SRMR lay below the respective thresholds. Therefore, scalar and residual invariance were only partially supported. Taken together, these results indicate that the proposed one-factor structure of the German DACS is acceptable for all subsamples.

**Table 7:** Standardized loadings for structural equation modeling for the DACS in different subsamples.

DACS item	Standardized loadings λ* for women without EDs (*n* = 1,360)	Standardized loadings λ* for men without EDs (*n* = 304)	Standardized loadings λ* for women with EDs (*n* = 450)
Item 1	0.70	0.73	0.60
Item 2	0.71	0.76	0.55
Item 3	0.70	0.67	0.65
Item 4	0.85	0.88	0.84
Item 5	0.71	0.63	0.66
Item 6	0.84	0.83	0.87
Item 7	0.90	0.91	0.90
Item 8	0.89	0.89	0.90

Structural equation modeling with maximum likelihood estimation. DACS, Downward Physical Appearance Comparison Scale. ED, eating disorder. The specific items can be derived from Table 3.

### 3.3 Internal consistency

For women without EDs (*n* = 1,360), the internal consistencies of the two scales were acceptable, at McDonald’s ω_t_ = 0.95 for the UPACS and McDonald’s ω_t_ = 0.94 for the DACS. Similarly, for men without EDs (*n* = 304), the internal consistencies were acceptable, at McDonald’s ω_t_ = 0.96 for the UPACS and McDonald’s ω_t_ = 0.95 for the DACS. For women with EDs (*n* = 450), internal consistency was acceptable for the UPACS, at McDonald’s ω_t_ = 0.93, and for the DACS, at McDonald’s ω_t_ = 0.94.

### 3.4 Test–retest reliability

The test–retest reliability could only be determined for participants who took part in the retest in Study 9 (*n* = 232). On average, the interval between the first and second assessment of the UPACS and DACS lay at *M* = 16.47 days (*SD* = 4.65). For women without EDs (*n* = 142), the test–retest reliability was *r*_tt_ = 0.86, *p* < 0.001 for the UPACS and *r*_tt_ = 0.70, *p* < 0.001 for the DACS. For men without EDs (*n* = 50), the test–retest reliability was *r*_tt_ = 0.80, *p* < 0.001 for the UPACS and *r*_tt_ = 0.64, *p* < 0.001 for the DACS. For women with EDs (*n* = 40), the test–retest reliability was *r*_tt_ = 0.84, *p* < 0.001 for the UPACS and *r*_tt_ = 0.71, *p* < 0.001 for the DACS.

### 3.5 Construct validity

In women and men without EDs, the UPACS and DACS showed significant positive correlations with each other, indicating that a higher tendency for upward appearance comparisons is associated with a higher tendency for downward comparisons, and vice versa (see Tables 8, 9). For women without EDs, the correlation between the UPACS and DACS was not significant (see Table 10).

As hypothesized, in women without EDs (see Table 8), the UPACS and DACS scores showed significant positive correlations with the PACS, the two subscales of the EDI-2 and the EDE-Q. The UPACS and DACS scores showed significant negative correlations with the RSES scores. All effect sizes were descriptively higher for the correlations with the UPACS than for the correlations with the DACS.

**Table 8:** Means, standard deviations, and correlations of the UPACS and DACS and related measures in women without eating disorders.

Scale	*n*	*M*	SD	UPACS	DACS
UPACS	1,360	3.30	0.90	–	0.31^***^
DACS	1,360	2.35	0.94	0.31^***^	–
PACS	554	2.84	0.79	0.73^***^	0.42^***^
EDI-2 BD	1,360	3.21	1.20	0.29^***^	0.23^***^
EDI-2 DFT	1,360	2.64	1.21	0.39^***^	0.27^***^
EDE-Q	1,360	1.42	1.17	0.36^***^	0.24^***^
RSES	1,360	2.15	0.60	−0.37^***^	−0.20^***^

UPACS, Upward Physical Appearance Comparison Scale; DACS, Downward Physical Appearance Comparison Scale; PACS, Physical Appearance Comparison Scale; EDI-2, Eating Disorder Inventory-2; BD, Body dissatisfaction; DFT, Drive for thinness; EDE-Q, Eating Disorder Examination Questionnaire; RSES, Rosenberg Self-Esteem Scale; M, mean; SD, standard deviation. ^*^p < 0.05, ^**^p < 0.01, ^***^p < 0.001 with Bonferroni correction.

In men without EDs, the correlations followed a similar pattern as in women without EDs (see Table 9). From a descriptive perspective, in men, the effect sizes for the correlations of the EDE-Q with the UPACS and DACS differed marginally. Furthermore, the effect size of the correlation of the EDI-2 subscale *Drive for thinness* with the DACS was slightly higher than that with the UPACS. Overall, all effect sizes were descriptively smaller in men than in women without EDs, indicating stronger associations of upward and downward appearance comparisons with related constructs in women than in men without EDs.

**Table 9:** Means, standard deviations, and correlations of the UPACS and DACS and related measures in men without eating disorders.

Scale	*n*	*M*	SD	UPACS	DACS
UPACS	304	2.90	0.94	–	0.41^***^
DACS	304	2.08	0.86	0.41^***^	–
PACS	78	2.56	0.78	0.59^***^	0.38^**^
EDI-2 BD	304	2.41	0.93	0.28^***^	0.20^**^
EDI-2 DFT	304	1.88	0.89	0.24^***^	0.27^***^
EDE-Q	304	0.93	0.85	0.27^***^	0.26^***^
RSES	304	2.33	0.55	−0.27^***^	−0.21^**^

UPACS, Upward Physical Appearance Comparison Scale; DACS, Downward Physical Appearance Comparison Scale; PACS, Physical Appearance Comparison Scale; EDI-2, Eating Disorder Inventory-2; BD, Body dissatisfaction; DFT, Drive for thinness; EDE-Q, Eating Disorder Examination Questionnaire; RSES, Rosenberg Self-Esteem Scale; M, mean; SD, standard deviation. ^*^p < 0.05, ^**^p < 0.01, ^***^p < 0.001 with Bonferroni correction.

A different pattern emerged in women with EDs (see Table 10). The correlations of the PACS, the EDI-2 subscale *Drive for thinness*, and the EDE-Q with the UPACS and DACS were in the expected direction, with stronger effect sizes for the UPACS. The RSES showed a significant negative correlation with the UPACS but not with the DACS. The EDI-2 subscale *Body dissatisfaction* was not significantly correlated with either the UPACS or the DACS.

**Table 10:** Means, standard deviations, and correlations of the UPACS and DACS and related measures in women with eating disorders.

Scale	*n*	*M*	SD	UPACS	DACS
UPACS	450	3.85	0.77	–	0.11
DACS	450	2.71	0.98	0.11	–
PACS	124	3.52	0.72	0.51^***^	0.34^**^
EDI-2 BD	450	4.86	0.91	0.08	0.10
EDI-2 DFT	450	4.87	0.92	0.35^***^	0.16^*^
EDE-Q	450	4.01	1.13	0.26^***^	0.17^**^
RSES	450	1.13	0.62	−0.30^***^	−0.05

UPACS, Upward Physical Appearance Comparison Scale; DACS, Downward Physical Appearance Comparison Scale; PACS, Physical Appearance Comparison Scale; EDI-2, Eating Disorder Inventory-2; BD, Body dissatisfaction; DFT, Drive for thinness; EDE-Q, Eating Disorder Examination Questionnaire; RSES, Rosenberg Self-Esteem Scale; M, mean; SD, standard deviation. ^*^p < 0.05, ^**^p < 0.01, ^***^p < 0.001 with Bonferroni correction.

## 4 Discussion

The aim of the present study was to investigate the German-language versions of the UPACS and DACS in different samples of women and men without EDs and women with EDs. Using data collected over nine studies, we examined the psychometric properties and factor structure of both scales.

Regarding the factor structure of the scales, the results of the SEM and tests of measurement invariance indicated that the one-factor structure of the original English-language version is adequate for the DACS but not for the UPACS in all subsamples. Exploratory re-specifications based on modification indices indicated that the poor fit could be due to two similar items, concerning upward appearance comparisons with “magazine models” and with “models and movie stars.” When allowing for correlation of the residuals of these two items, the fit of the one-factor model improved noticeably, suggesting that these items are closely interrelated, possibly due to the similar wording of the items. As both items include comparisons with models, with one specifying comparisons with magazine models, it is evident that the two items heavily overlap in content and in item wording. Moreover, it is questionable whether comparisons with models and celebrities like movie stars have the same qualities as they did back in 2009, when the original scale was developed, as the rise of social media since that time has led to a new type of comparisons, namely with so-called influencers (Ye et al., 2021). Nowadays, people tend to compare themselves with celebrities and influencers on social media rather than with models seen in traditional media, especially magazines or billboard advertisements (Fardouly et al., 2017), and may therefore identify themselves more with influencers than with other celebrities (Schouten et al., 2020). To account for this change, Roberts et al. (2022) adapted the tripartite influence model (Thompson et al., 1999) and identified social media as a separate factor influencing body dissatisfaction apart from traditional media. Additionally, the standardized loadings and item-total correlations of the two items in question were descriptively the smallest in each subsample, indicating that they are not associated as strongly with the underlying factor or the rest of the scale, respectively. Therefore, future studies should consider adapting the wording to the modern context or the removal of these two items. Until then, the complete version of the UPACS should only be used while taking into account the possible limitations regarding the factor structure and the possibly outdated item wording.

The internal consistencies were acceptable for both scales in all examined subsamples. The item-total correlations indicate that each item correlates sufficiently with each respective scale. Furthermore, the test–retest reliability was good in all examined subsamples. Taken together, the reliability of the UPACS and DACS can be considered adequate.

Additionally, women without EDs showed significantly higher scores on the UPACS and DACS than did men without EDs, which was expected due to women’s greater tendency to engage in appearance comparisons (Davison and McCabe, 2005; Strahan et al., 2006). Moreover, in line with expectations, women with EDs scored significantly higher on both scales than did women without EDs, which confirms the higher tendency for appearance comparisons in women with EDs (Blechert et al., 2009; Arigo et al., 2014). The findings indicate that the UPACS and DACS are able to detect differences in the tendency for upward and downward appearance comparisons.

Regarding the construct validity, the correlation patterns differed between the examined subsamples. It is important to note that the following discussion is based on descriptive effect sizes, as the correlations were not tested against each other. A further study evaluating the construct validity and including further inferential statistical data analyses is needed. Nevertheless, the correlation patterns provide a first overview indicating the construct validity of the scales. Most importantly, in each subsample, the UPACS and DACS correlated with the PACS. This is in line with expectation, as the PACS assesses general appearance comparisons and is therefore closely connected to the constructs of upward and downward physical appearance comparisons. In accordance with this, the effect sizes of the correlations with the PACS were descriptively higher than the effect sizes of the correlations with other related measures in each subsample. As expected, the effect size for the correlation between the UPACS and PACS was descriptively higher than that between the DACS and PACS, as upward appearance comparisons are generally more prevalent than downward appearance comparisons (Ridolfi et al., 2011; McCarthy et al., 2023) and should therefore be more closely related to the general tendency of making appearance comparisons. Accordingly, these results indicate that the scales do, in fact, assess appearance comparisons.

For women without EDs, the correlation patterns followed the overall expectations regarding positive associations with eating pathology, body dissatisfaction, and drive for thinness and negative associations with self-esteem. Furthermore, all effect sizes were descriptively higher for the correlations of the UPACS compared to those of the DACS, supporting the more conclusive and closer relationship of upward appearance comparisons with body image disturbance (Leahey et al., 2007). Findings regarding the influence of downward appearance comparisons on body image, by contrast, are inconsistent, with some studies reporting a positive influence (van den Berg and Thompson, 2007; Bailey and Ricciardelli, 2010) and others showing no positive effects, or even detrimental effects (Lin and Soby, 2016; Fitzsimmons-Craft, 2017; Drutschinin et al., 2018). The present study tends to support the latter findings.

For men without EDs, the UPACS and DACS showed positive correlations with the measures of eating pathology, body dissatisfaction, and drive for thinness and negative correlations with self-esteem. The pattern of effect sizes was not as conclusive as for women without EDs, as descriptively, the effect sizes for the correlations with the EDE-Q only differed marginally between the UPACS and DACS, and the effect size for the correlation of the DACS with the EDI-2 subscale *Drive for thinness* was descriptively slightly smaller than the correlation of the UPACS with this subscale. The similar effect sizes for the correlations with the UPACS and DACS might be due to the overall low scores and the limited variability on the EDE-Q and EDI-2 subscale *Drive for thinness* in men without EDs, because men generally show lower eating disorder pathology than women (Smink et al., 2012) and because thinness might be less important for evaluating one’s own appearance in men compared to women (Anderson and Bulik, 2004).

For women with EDs, the lack of association of both the UPACS and DACS with the EDI-2 subscale *Body dissatisfaction* might be due to a lack of variance in the subscale. The distribution of scores on the EDI-2 is left-skewed in this subsample, since most women with EDs show high body dissatisfaction *per se*, given that body dissatisfaction is a prevalent risk factor for EDs (Grabe et al., 2008; Rohde et al., 2015). The RSES only showed a negative correlation with the UPACS and not with the DACS, which might indicate that comparisons with people who are perceived to look better have a greater impact on self-esteem in women with EDs. This finding might be attributable to negative cognitive biases in women with EDs, which lead to more body checking behavior (Williamson et al., 2004) that possibly encompasses more upward appearance comparisons with women perceived to be more attractive than oneself as compared to downward comparisons.

Several limitations of the present study should be mentioned. First, the initial translation process did not include a back-translation by a native English speaker; thus, complete correspondence between the original version and the German version of the UPACS and DACS cannot be fully ensured. However, a bilingual translator did subsequently compare the two versions and found only three minor expressions that could have been altered but would not have meaningfully changed the item contents. This suggests that the translation can be deemed as acceptable. Second, the data are derived from nine different studies with varying study aims, designs, and target populations, conducted at different time points between 2016 and 2023. While all studies included the same German-language version of the UPACS and DACS, the studies differed in length and the questionnaires were administered at different positions within the studies. Therefore, it cannot be ensured that certain preceding questionnaires or contents in the respective studies influenced participants’ responses to the UPACS and DACS. Third, in three of the studies, EDs were not diagnosed with a structured clinical interview, but rather relied on participants’ self-reports. It is possible that some participants may have provided a false or only presumed diagnosis. However, compared to participants with EDs diagnosed as part of a study, those with self-reported EDs had comparable or even higher scores on relevant questionnaires regarding ED pathology, suggesting that the majority of self-reported diagnoses were appropriate for the category of EDs. It might be the case that individuals with higher levels of ED pathology were more likely to participate in studies in which self-reported diagnoses were sufficient because the procedure was less stressful, more anonymous, and the studies were easier to access. Fourth, the psychometric properties and factor structure of the UPACS and DACS were examined for women with EDs in general rather than separately for women with anorexia nervosa, bulimia nervosa, and binge eating disorder. Despite some overlap in the characteristics of the different EDs, e.g., possible binge-eating episodes, the EDs differ, for instance, regarding weight and compensatory behavior (American Psychiatric Association, 2013). We chose to analyze all EDs within one group to enable sufficient sample sizes, especially for the SEM analyses and the assessment of test–retest reliability. However, future studies should examine the validity of the scales for each form of ED separately. Fifth, due to the lack of men with eating disorders and non-binary people in the final sample, no statement can be made regarding the suitability of the UPACS and DACS for assessing upward and downward appearance comparisons in these two groups. Future evaluations of the scales should therefore endeavor to include these groups. Finally, we only examined questionnaires that were related to the concepts of appearance comparisons in some way, thus focusing most on convergent validity. To ensure divergent validity, future studies should encompass more measures assessing variables that are less or not related to appearance comparisons.

Despite these limitations, the present study is the first to evaluate German-language versions of the UPACS and DACS, and might thus constitute a first step towards enabling the use of both scales in future research. Overall, the reliability and validity of the German-language UPACS and DACS are satisfactory, with limitations especially regarding the factor structure of the UPACS. Moreover, as the data were collected across nine studies, it was possible to examine the properties of the UPACS and DACS in women and men without EDs as well as women with EDs with sufficient sample sizes.

Future studies should seek to refine the scales, especially the UPACS, to account for modern-day developments in comparison processes or to generate a more general assessment of appearance comparisons that can be used outside the context of the rapidly changing media environment. The proposed re-specifications of the UPACS were only examined for exploratory purposes and need to be confirmed in independent samples. As the data were derived from several different studies, a future study should be designed and conducted solely for the purpose of validating the UPACS and the DACS.

Overall, the German-language versions of the UPACS and DACS seem to be useful scales to assess upward and downward appearance comparisons in women and men without EDs and women with EDs. Differentiating the direction of appearance comparisons allows for a more specific examination of the influence of appearance comparisons on body image and related constructs and as a risk factor for EDs. In sum, this study is the first to indicate that the German-language UPACS and DACS might be suitable for use in research and clinical practice.

## Data availability statement

The datasets presented in this article are not readily available because the informed consent in four of the studies (Voges et al., 2018, 2019, 2020; Quittkat et al., 2023) excludes the distribution of data to third parties, so that only the data from the remaining five study waves can be made available upon request. Requests to access the datasets should be directed to kristine.schoenhals@uni-osnabrueck.de.

## Ethics statement

The studies involving humans were approved by the Ethics committee of Osnabrück University. The studies were conducted in accordance with the local legislation and institutional requirements. The participants provided their written informed consent to participate in this study.

## Author contributions

KS: Conceptualization, Formal analysis, Investigation, Methodology, Project administration, Visualization, Writing – original draft. HQ: Conceptualization, Investigation, Writing – review & editing. MV: Investigation, Writing – review & editing. GL: Investigation, Writing – review & editing. F-JH: Investigation, Writing – review & editing. SV: Conceptualization, Resources, Supervision, Writing – review & editing.
