# Supplementary material for: Is my body better than yours? Validation of the German version of the Upward and Downward Physical Appearance Comparison Scales in individuals with and without eating disorders
Source: Front Psychol. 2024 May 28;15:1390063. doi: 10.3389/fpsyg.2024.1390063 (PMC11186468; doi:10.3389/fpsyg.2024.1390063)
Supplement: Supplementary file 1 [file Data_Sheet_1.docx]

Supplementary Material

**German version of the Upward and Downward Physical Appearance Comparison Scales (UPACS and DACS)**

Hier finden Sie ein paar Fragen zu Vergleichen mit anderen Menschen hinsichtlich des Aussehens. Bitte schätzen Sie ein, wie häufig Sie die folgenden Verhaltensweisen zeigen. Es gibt keine richtigen oder falschen Antworten.

|  | stimme überhaupt nicht zu | stimme eher nicht zu | weder-noch | stimme eher zu | stimme ausgesprochen zu |
| --- | --- | --- | --- | --- | --- |
| 1. Ich vergleiche mich eher mit Personen, die besser aussehen als ich, als mit solchen, die schlechter aussehen. | 1 | 2 | 3 | 4 | 5 |
| 1. Ich neige dazu, meine körperliche Attraktivität mit der von Zeitschriftenmodels zu vergleichen. | 1 | 2 | 3 | 4 | 5 |
| 1. Wenn ich eine Person sehe, die körperlich unattraktiv ist, denke ich darüber nach, wie ich im Vergleich zu ihr dastehe. | 1 | 2 | 3 | 4 | 5 |
| 1. Ich neige dazu, meinen Körper mit denen von Personen zu vergleichen, die einen unterdurchschnittlichen Körper haben. | 1 | 2 | 3 | 4 | 5 |
| 1. Ich denke darüber nach, ob mein eigenes Aussehen mit dem Aussehen von Models und Filmstars vergleichbar ist. | 1 | 2 | 3 | 4 | 5 |
| 1. Am Strand oder beim Sport vergleiche ich meinen Körper mit denen von Personen, die einen weniger athletischen Körper haben. | 1 | 2 | 3 | 4 | 5 |
| 1. Am Strand oder beim Sport frage ich mich, ob mein Körper genauso attraktiv ist wie der von den Personen, die einen sehr attraktiven Körper haben. | 1 | 2 | 3 | 4 | 5 |
| 1. Ich vergleiche mich mit Personen, die weniger gut aussehen als ich. | 1 | 2 | 3 | 4 | 5 |
| 1. Ich neige dazu, mich mit Personen zu vergleichen, von denen ich denke, dass sie besser aussehen als ich. | 1 | 2 | 3 | 4 | 5 |
| 1. Ich denke darüber nach, wie attraktiv mein Körper im Vergleich zu denen von übergewichtigen Personen ist. | 1 | 2 | 3 | 4 | 5 |
| 1. Wenn ich jemanden mit einem tollen Körper sehe, frage ich mich, wie ich im Vergleich zu dieser Person abschneide. | 1 | 2 | 3 | 4 | 5 |
| 1. Auf Partys vergleiche ich oft mein Aussehen mit dem Aussehen von unattraktiven Personen. | 1 | 2 | 3 | 4 | 5 |
| 1. Ich vergleiche mich oft mit Personen die körperlich weniger attraktiv sind. | 1 | 2 | 3 | 4 | 5 |
| 1. Wenn ich gutaussehende Personen sehe, frage ich mich, wie ich im Vergleich zu ihnen dastehe. | 1 | 2 | 3 | 4 | 5 |
| 1. Auf Partys oder anderen gesellschaftlichen Veranstaltungen vergleiche ich mein Aussehen mit dem Aussehen von sehr attraktiven Personen. | 1 | 2 | 3 | 4 | 5 |
| 1. Ich neige dazu, mein Aussehen mit dem Aussehen von Personen zu vergleichen, deren Körper weniger ansprechend sind als meiner. | 1 | 2 | 3 | 4 | 5 |
| 1. Ich vergleiche mein Aussehen mit dem von Personen, die besser aussehen als ich. | 1 | 2 | 3 | 4 | 5 |
| 1. Ich vergleiche meinen Körper mit dem von Personen, die einen besseren Körper haben als ich. | 1 | 2 | 3 | 4 | 5 |

UPACS: Items 1, 2, 5, 7, 9, 11, 14, 15, 17, 18

DACS: Items 3, 4, 6, 8, 10, 12, 13, 16

**Table S1.** Skewness and kurtosis of the UPACS items in different subsamples

| UPACS item | Women without EDs  (*n* = 1,360) | | Men without EDs  (*n* = 304) | | Women with EDs  (*n* = 450) | |
| --- | --- | --- | --- | --- | --- | --- |
|  | Skewness | Kurtosis | Skewness | Kurtosis | Skewness | Kurtosis |
| 1. | -0.80 | 0.29 | -0.80 | 0.22 | -1.24 | 1.46 |
| 2. | 0.50 | -1.07 | 1.14 | 0.25 | -0.07 | -1.18 |
| 3. | 0.22 | -1.36 | 0.57 | -0.98 | -0.13 | -1.37 |
| 4. | -0.62 | -0.81 | -0.39 | -1.16 | -0.84 | -0.45 |
| 5. | -1.07 | 0.46 | -0.60 | -0.78 | -1.64 | 2.84 |
| 6. | -0.79 | -0.34 | -0.39 | -1.17 | -1.55 | 2.30 |
| 7. | -0.88 | -0.19 | -0.40 | -1.12 | -1.54 | 2.50 |
| 8. | -0.71 | -0.60 | -0.27 | -1.26 | -1.29 | 1.22 |
| 9. | -1.01 | 0.22 | -0.54 | -0.97 | -1.56 | 2.63 |
| 10. | -0.99 | 0.17 | -0.65 | -0.80 | -1.63 | 3.12 |

*Note.* UPACS = Upward Physical Appearance Comparison Scale; ED = eating disorder.

**Table S2.** Skewness and kurtosis of the DACS items in different subsamples

| DACS item | Women without EDs  (*n* = 1,360) | | Men without EDs  (*n* = 304) | | Women with EDs (*n* = 450) | |
| --- | --- | --- | --- | --- | --- | --- |
|  | Skewness | Kurtosis | Skewness | Kurtosis | Skewness | Kurtosis |
| 1. | -0.13 | -1.33 | 0.17 | -1.38 | -0.55 | -0.85 |
| 2. | 0.51 | -0.76 | 0.90 | 0.08 | 0.14 | -1.04 |
| 3. | 0.28 | -1.18 | 0.66 | -0.46 | 0.18 | -1.18 |
| 4. | 0.41 | -0.98 | 0.80 | -0.04 | 0.38 | -0.90 |
| 5. | 0.35 | -1.26 | 0.67 | -0.84 | -0.12 | -1.36 |
| 6. | 0.81 | -0.31 | 0.97 | 0.38 | 0.43 | -0.93 |
| 7. | 0.66 | -0.62 | 0.99 | 0.28 | 0.47 | -0.94 |
| 8. | 0.56 | -0.85 | 0.89 | -0.06 | 0.39 | -0.95 |

*Note.* DACS = Downward Physical Appearance Comparison Scale; ED = eating disorder.

**Figure S1.** Box plots for the UPACS scores in the different subsamples.

**
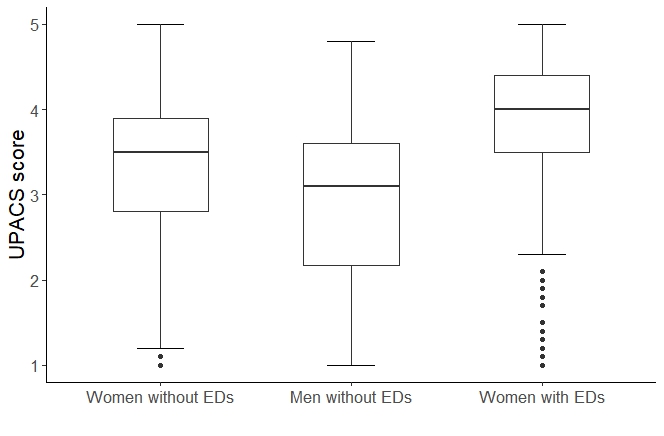
**

*Note*. UPACS = Upward Physical Appearance Comparison Scale; ED = eating disorder.

**Figure S2.** Box plots for the DACS scores in the different subsamples.

**
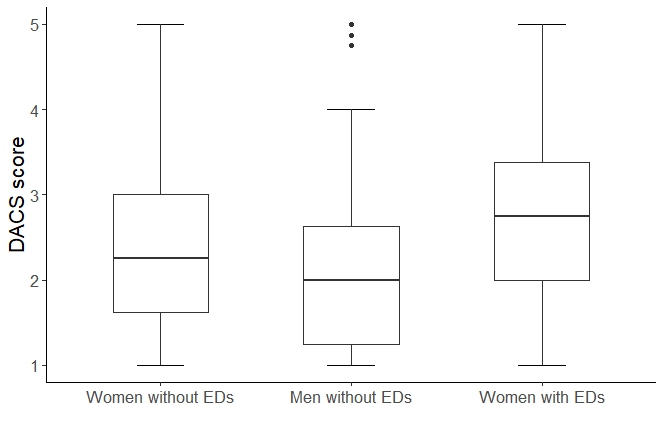
**

*Note.* DACS = Downward Physical Appearance Comparison Scale; ED = eating disorder.
